# Epistemonikos

**DOI:** 10.5195/jmla.2017.260

**Published:** 2017-10-01

**Authors:** Yamila M. El-Khayat

Created in 2009 at Pontificia Universidad Católica de Chile, Epistemonikos is designed to allow researchers to search evidence-based medical literature using a structured patient or problem, intervention, comparison, outcome (PICO) question to facilitate clinical decision making. The Epistemonikos Foundation mentions that the main goal for this resource is “to bring evidence closer to those making health decisions, through technology and innovation” [1].

This resource was built to be a repository of all existing evidence related to a health question, allowing users to search for systematic reviews and related material, including but not limited to primary studies included in systematic reviews, overviews of reviews, evidence-based policy briefs and guidelines based on reviews, and structured summaries of evidence presented in reviews. The creators of Epistemonikos, plus over 250 collaborators, monitor 30 databases regularly to populate the resource [2, 3], including Cochrane, PubMed, EMBASE, and CINAHL. According to the Epistemonikos website, 3 categories of articles are considered for inclusion: broad synthesis (articles synthesizing systematic reviews or, sometimes, primary studies), synthesis of primary studies, and primary studies [4].

Epistemonikos now holds more than 115,000 unique documents, and more than 100,000 relationships between the documents are stored and indexed. Once a unique piece is identified, it is forwarded to an Epistemonikos expert for inclusion in the database.

## ARTICLE TRANSLATIONS

Epistemonikos is available in nine languages: Arabic, Chinese, Dutch, English, French, Italian, German, Portuguese, and Spanish. All certified translations of a systematic review are linked to the review itself. These are marked as “official translation.” In other cases, collaborators and domain experts translate articles themselves; these are labeled as “collaborative translations.” Many articles are also translated using automated tools like Google Translate; because these translations are not necessarily free of error, these are marked as “automatic translations.”

## DATABASE SEARCHING

Epistemonikos offers simple and advanced searches. The simple search is available via a search box on the home page. Here, you can enter a term for a condition or intervention; there is no need for Boolean logic, as the database automatically connects terms. The advanced search allows you to restrict searches to title, authors, or abstract. A search history is also available, which allows you to combine searches. The advanced search supports truncation, exact phrase searching, and Boolean operators. Both simple and advanced searches can be conducted in any of the nine languages that the database supports.

Search results are presented in order of relevance using a rubric based on the frequency with which the search term appears. Results are color coded to indicate whether they are systematic reviews, primary studies, structured summaries of systematic reviews, or broad syntheses. If the search topic has similar subtopics that occur in several of the articles, a “Matrix of Evidence” will appear on the top of the search results.

If you create a personal account (at no charge), you can save documents, searches, and matrixes. Matrixes provide a visualization of the systematic reviews and the primary studies that are available related to a reference.

## SEARCH OPTIONS

Once you search, filters are available to narrow your results by category (broad synthesis, systematic review, structured summaries, or primary summaries) or by year (last year, last 5 years, last 10 years, or custom range). Smart filters are automatically applied to remove less relevant results; they can be turned off by clicking on the On/Off button, located directly below the filters.

**Figure 1:**
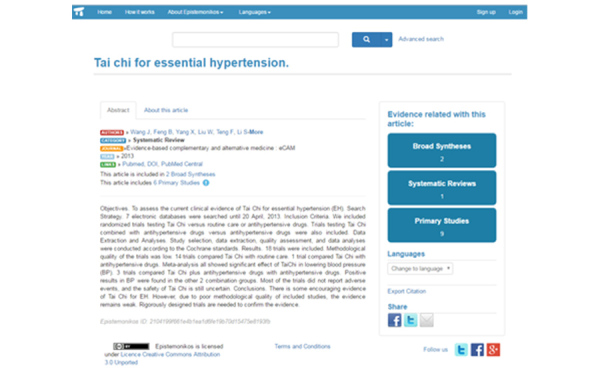
Article from search results for “Tai chi for essential hypertension”

Figure 1 shows a systematic review related to hypertension. In this screen, you can see the citation and abstract, along with the number of broad syntheses, systematic reviews, and primary studies related to this article. You can also change the language of this article by clicking on the drop-down menu below these.

## ADDITIONAL FEATURES

On the home page of Epistemonikos, next to the search bar, a small window presents a live Twitter feed of what is being said about this resource and topics that it covers. Epistemonikos has a strong social media presence, with accounts you can follow on Twitter, Facebook, and Google+. If you search Epistemonikos on Pinterest, you will also find quite a few results.

Epistemonikos seems to work well on current versions of all major web browsers and mobile devices, including phones and other devices with small screens.

## COMPARISON WITH OTHER DATABASES

Epistemonikos has been compared many times to the Cochrane Library. Strømme et al. note that there are many similarities between these resources, including many articles that overlap, but both have unique features [5]. What makes Epistemonikos stand out is its support for multiple languages, which makes it more user friendly globally.

## CONCLUSION

Epistemonikos is a succinct, complete, and multilingual database that allows users to find information quickly for clinical decision-making. Because most of the literature, especially systematic reviews, is published in English, this resource is a powerful tool for non-English speakers to find systematic reviews, read the literature, and understand concepts presented in their native languages.
